# A Sensitive A_3_B Porphyrin Nanomaterial for CO_2_ Detection

**DOI:** 10.3390/molecules191221239

**Published:** 2014-12-17

**Authors:** Eugenia Fagadar-Cosma, Dana Vlascici, Gheorghe Fagadar-Cosma, Anca Palade, Anca Lascu, Ionela Creanga, Mihaela Birdeanu, Rodica Cristescu, Ileana Cernica

**Affiliations:** 1 Department of Organic Chemistry, Institute of Chemistry Timisoara of Romanian Academy, M. Viteazul Ave, No. 24, 300223 Timisoara, Romania; E-Mails: danavlascici@yahoo.com (D.V.); anca_palade@yahoo.com (A.P.); ancalascu@yahoo.com (A.L.); ionelacreanga@yahoo.com (I.C.); mihaione2002@yahoo.com (M.B.); 2 Department of Chemistry, Chemistry-Biology-Geography Faculty, West University of Timisoara, Pestalozzi Street, No. 16, 300115 Timisoara, Romania; 3 CAICAM Department, Faculty of Industial Chemistry and Environmental Engineering, Politehnica University, V. Parvan Bv., No. 6, 300223 Timisoara, Romania; E-Mail: gfagadar@yahoo.com; 4 National Institute for Research and Development in Electrochemistry and Condensed Matter, P. Andronescu Street, No. 1, 300224 Timisoara, Romania; 5 Lasers Department, National Institute for Lasers, Plasma & Radiation Physics, P.O. Box MG-36, Magurele, 077125 Bucharest, Romania; E-Mail: rodica_cristescu@yahoo.com; 6 National R&D Institute for Microtechnology, Erou Iancu Nicolae Street, No. 126 A, Voluntari, 077190 Bucharest, Romania; E-Mail: ileana.cernica@imt.ro

**Keywords:** asymmetric A_3_B porphyrins, UV-vis spectroscopy, sensitivity to CO_2_, self-assembling, AFM

## Abstract

The present report deals with the tailoring, preparation and characterization of novel nanomaterials sensitive to CO_2_ for use in detection of this gas during space habitation missions. A new nanostructured material based on mixed substituted asymmetrical A_3_B porphyrin: 5-(4-pyridyl)-10,15,20-tris(3,4-dimethoxyphenyl)-porphyrin (PyTDMeOPP) was synthesized and characterized by ^1^H-NMR, FT-IR, UV-vis, fluorescence, MS, HPLC and AFM. Introducing one pyridyl substituent in the 5-*meso*-position of porphyrin macrocycle confers some degree of hydrophilicity, which may cause self-assembly properties and a better response to increased acidity. The influence of pH and nature of the solvent upon H and J aggregates of the porphyrin are discussed. Porphyrin aggregation at the air–THF interface gave a triangular type morphology, randomly distributed but uniformly oriented. When deposition was made by multiple drop-casting operations, a network of triangles of uniform size was created and a porous structure was obtained, being reorganized finally in rings. When the deposition was made from CHCl_3_, ring structures ranging in internal diameter from 300 nm to 1 µm, but with the same width of the corona circular of approx. 200 nm were obtained. This porphyrin-based material, capable of generating ring aggregates in both THF and CHCl_3_, has been proven to be sensitive to CO_2_ detection. The dependence between the intensity of porphyrin UV-vis absorption and the concentration of CO_2_ has a good correlation of 98.4%.

## 1. Introduction

Spectrophotometric and colorimetric microsensors for an easy and friendly detection of the air quality (*i.e.*, detection of CO, NO_x_ and high level of CO_2_ and low level of O_2_) have attracted much interest in the last decade. Optical CO_2_ sensors based on color changes of the pH indicator dye α-naphthol-phthalein coupled with the fluorescence of tetraphenylporphyrin attached to a polystyrene layer have been intensively studied on different substrates, but especially on ethyl cellulose layers [1,2,3,4,5]. The response times of the sensing film in all investigated cases were less than 5.0 s for switching from nitrogen to CO_2_, and for switching from CO_2_ to nitrogen. Besides nitrogen, the authors also used argon as neutral gas. The detection of CO_2_ in water was also attempted using the same combination [6].

Another reported porphyrin derivative used in combination with α-naphtholphthalein as colorimetric pH indicator in order to detect CO_2_ is a platinum porphyrin dye (Pt-tetrakispentafluorophenylporphyrin) incorporated in a plastic matrix together with a phase transfer agent tetraoctyl- or cetyltrimethyl-ammonium hydroxide [7]. CO_2_ sensors which use an inner filter quenching effect were also described. One such system consisted of a phosphorescent platinum octaetylporphyrin and a pH-sensitive dye in poly(vinylidene chloride-co-vinyl chloride-ethyl cellulose) thin films or microparticles with low permeability to oxygen [8]. A portable instrument to measure CO_2_ in the gas phase [9] based on a solid-state sensor film, where the platinum octaethylporphyrin complex is quenched by the deprotonated form of naphtholphthalein [10] was also reported.

Sensing of other toxic gases such as NO_2_, NH_3_, CO and H_2_S has been performed using Langmuir-Blodgett thin films of several Co, Fe, Cu, Ni metalloporphyrins, showing that the complexed inner metal is important for the selectivity of the sensor.

New sensors based on Mn(III)-porphyrins and single wall carbon nanotubes (SWCNTs) embedded in barium stearate multilayers realized by the Langmuir-Blodgett (LB) technique have been tested for sensitivity to NO_2_ gas. A sensitive effect of the sensor for NO_2_ gas was demonstrated. The coverage of the sensor with a metalloporphyrin (Mn) leads to strong enhancement of the sensitive effect to NO_2_, at a few ppm, but the proposed material was not sensitive to the other toxic investigated gases, such as CO and CH_4_ besides, the selectivity was preserved both around room temperature and at higher operating temperatures (up to 200 °C) [11].

The importance of the morphology and topography of the thin film was put into evidence in another experiment, based on the same Mn(III)-porphyrin, but realized by the MAPLE technique. In this case the thin film was sensitive to neurotransmitters [12].

A dimer derivative of Ni(II)-octaethylporphyrin also deposited by the LB technique was selectively sensitive to NO gas in the presence of other interfering gases such as NH_3_ and CO. A few of these sensors are resistive ones [13]. 

Polypyrrole was chemically functionalized with 5,10,15,20-tetraphenyl-21*H*,23*H*-porphyrin iron(III) chloride (FeTPPCl) with special interest in detecting noxious carbon monoxide (CO) gas at ppm levels. The response of the material on the resistive sensor towards CO was quite fast and reversible [14,15].

The sodium salt of [5,10,15,20-tetra(4-sulphonatophenyl)-porphyrinato]zinc deposited on ZnO rods is sensitive to ppm concentrations of amines and alcohols [16]. The electron transport characteristics of Fe-porphyrin (FeP) and O_2_ adsorbed Fe-porphyrin (FeP-O_2_) have been investigated by the nonequilibrium Green’s function approach in combination with density functional theory. Recently, chlorophyll fluorescence was used for monitoring CO_2_ uptake by earth vegetation [17].

## 2. Results and Discussion

**Figure 1:**
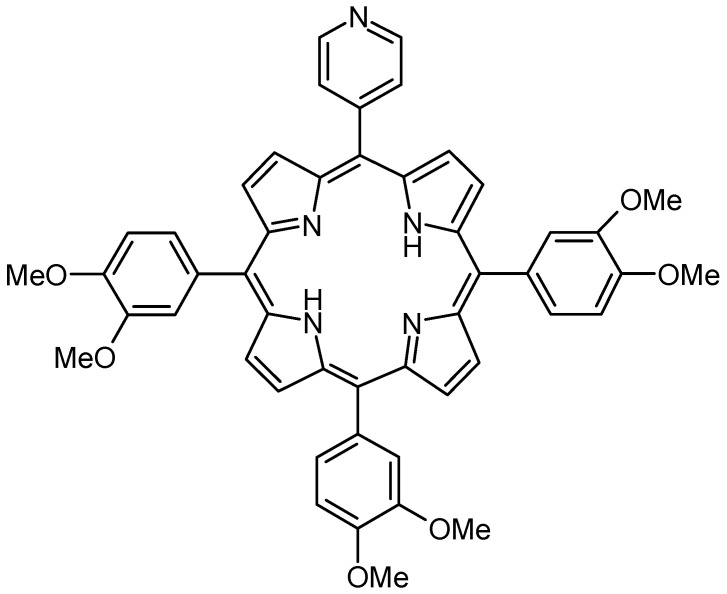
Structure of 5-(4-pyridyl)-10,15,20-tris(3,4-dimethoxyphenyl)porphyrin.

This work is focused on the preparation and further applications of 5-(4-pyridyl)-10,15,20-tris(3,4-dimethoxyphenyl)porphyrin (PyTDMeOPP) for the easy detection of CO_2_ gas in space habitable areas. The structure of (PyTDMeOPP) is presented in Figure 1.

### 2.1. IR Spectrum of the A_3_B Porphyrin 

The main features of the IR spectrum of the A_3_B porphyrin are the weak band located at 3311 cm^−1^ assigned to the ν N-H vibration, probably due to the fact that NH groups are involved in intramolecular H-bonding, and the sharp and intense band at 1232 cm^−1^ corresponding to C-O-CH_3_ vibrations. 

### 2.2. ^1^H-NMR Spectrum of the Porphyrin (PyTDMeOPP)

The ^1^H-NMR spectrum of the porphyrin (PyTDMeOPP) presents a signal corresponding to the internal NH pyrrolic protons that appears as broad singlet at −2.78 ppm and the signals for β-pyrrolic protons, which are located at 8.91 and 8.81 ppm. The signals for the 3,5-pyridyl protons are to be seen as a singlet at 8.20 ppm and those for 5-phenyl as a multiplet at 7.78–7.80. As expected, two singlet signals located at 4.18 and 3.99 ppm are attributed to the 4-methoxy- and 3-methoxyphenyl groups, respectively.

### 2.3. UV-Vis Spectrum of Compound (PyTDMeOPP)

**Figure 2:**
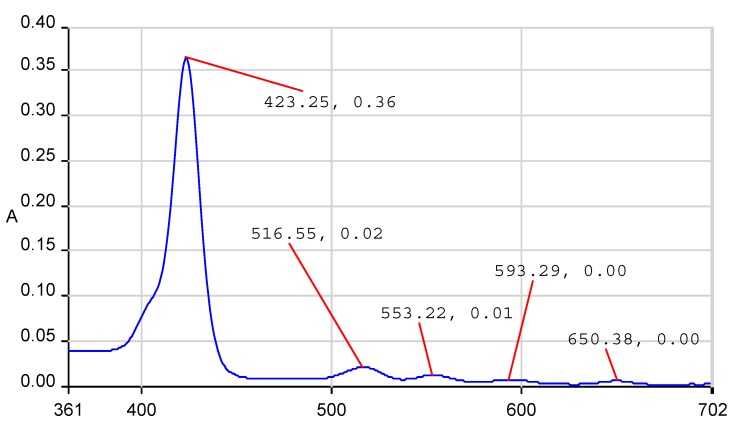
The UV-vis spectrum of 5-(4-pyridyl)-10,15,20-tris(3,4-dimethoxyphenyl)porphyrin.

The UV-vis spectrum of compound (PyTDMeOPP) displays the typical very intense Soret band accompanied by four Q-bands in the visible region (Figure 2). The Soret band, located at 423 nm, is attributed to the transition from a_1u_ (π) → e_g_* (π) and the other four absorption maxima around 516, 553, 593 and 650 nm are assigned to the Q bands corresponding to a_2u_ (π) → e_g_* (π) transitions. The UV-vis spectrum shows etio type shape, in which the intensity of the Q bands are decreasing in the order QIV > QIII > QII > QI. 

### 2.4. The Influence of the Acid Environment on UV-Vis Spectra

In order to be further used for CO_2_ sensing, 5-(4-pyridyl)-10,15,20-tris(3,4-dimethoxyphenyl)-porphyrin was subjected to some tests regarding its behavior in acidic environments. It is already known that most of the porphyrin bases manifest pronounced aggregation in acidic aqueous solutions, accompanied by changes regarding the position, the shape and the intensity of the main absorption bands [18].

**Figure 3:**
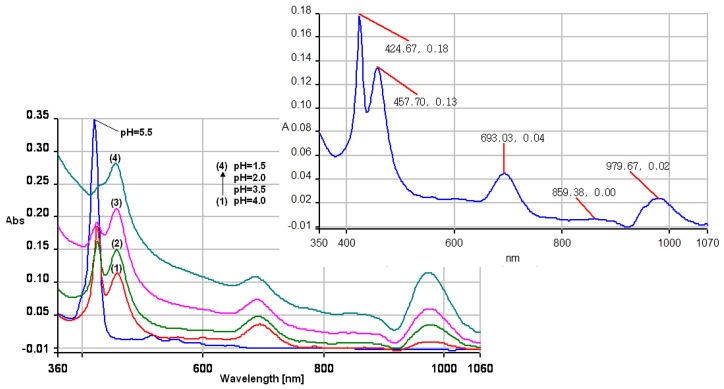
The superposed UV-vis spectra of 5-(4-pyridyl)-10,15,20-tris(3,4-dimethoxyphenyl)porphyrin, in methanol, pH = 5.5 (blue line), and in methanol-water systems with different amounts of HCl 0.1 N, curves 1 to 4. In detail curve 1.

When the acidity is increased to pH = 3 and lower, more additional protons are bonded both to the nitrogen atoms in the center of porphyrin ring, but also on the external pyridyl group. A high ionic strength causes a decrease of the monomer absorption Soret band situated around 420 nm, eventually reducing it to a pronounced shoulder. These changes in spectral shape (Figure 3) are associated with significant increase of the intensity of the QI band, but also with generation of a new band located around 970 nm. The QI band is hyperchromic and also bathochromically shifted from 650 nm (pH = 5) to 690 nm. Besides, the introduction of electron donating groups at the *meso*-positions raises the energy of the a_2u_ orbital by increasing its electron density and leads to the red shift of the Q (0,0) band [19], as confirmed in the UV-vis experiments.

With the increased ionic strength of the solution, a clear tendency to form J-aggregates is noticed. The band centered at 420 nm can be unequivocally assigned to the monomer and J-aggregates are characterized by a broadened red-shifted absorption band [20,21,22].

### 2.5. Excitation and Emission Spectra

**Figure 4:**
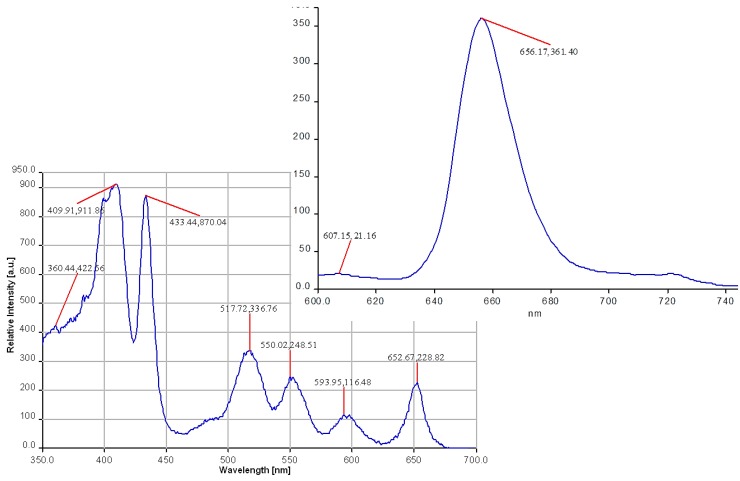
Excitation and emission spectra of 5-(4-pyridyl)-10,15,20-tris(3,4-dimethoxyphenyl)porphyrin, in THF, at λ_em_ = 603 nm (for excitation) and λ_ex_ = 420 nm (for emission).

From the excitation spectra (Figure 4), recorded at a higher concentration of porphyrin (10^−4^ mol/L), it is to be noted that the main peak is split into two peaks displaying a red-shift (433 nm) or a blue-shift (409 nm). This is a well-known behavior of porphyrins that initiate molecular association processes (J and H type aggregation) by increasing the concentration of the solution over 10^−6^ mol/L. The shape of the excitation spectra is in agreement with the exciton theory, which predicts that the excited-state energy level of a monomeric porphyrin splits in the course of aggregation. The hypsochromic shifted band located at 409 nm puts into evidence the generation of sandwich-type aggregates (H-aggregates) while the bathochromic shifted branch located at 433 nm is attributed to a side-by-side association process, due to generation of J-aggregates.

The emission spectra of bare porphyrin 2 in THF exhibited two maxima, a strong and broad Q_x_ (0,0) fluorescence band shifting from 656 nm and a weaker emission band situated at 725 nm, assigned to Q_x_ (0,1) transition. 

### 2.6. Atomic Force Microscopy Studies

It is unanimously accepted that the porphyrin macrocycle represents a favorite candidate for self-assembly and this can be realized by hydrogen bonding, π-π stacking interactions or hydrophobic effects and also by weak Van der Waals forces [21,23]. Besides, as a function of their tailored amphiphilic structure, A_3_B porphyrins can influence the size and shape of the nanoparticles [24,25] generating highly organized nanostructures [26,27,28].

**Figure 5:**
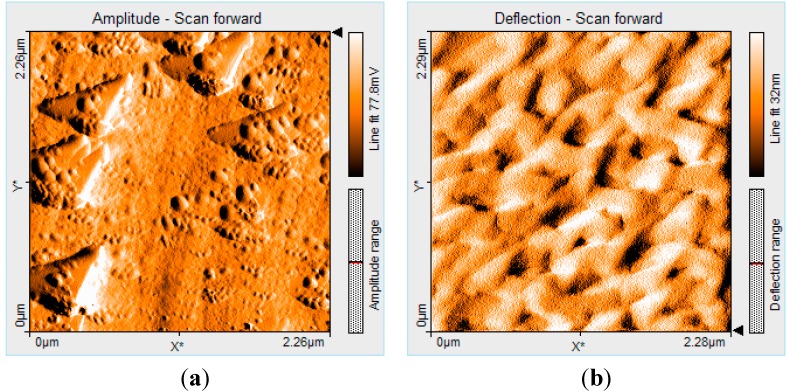
2D AFM image of 5-(4-pyridyl)-10,15,20-tris(3,4-dimethoxyphenyl)porphyrin (2.2 × 2.2 µm) from THF by drop-casting: (**a**) single and (**b**) multiple deposition.

**Figure 6:**
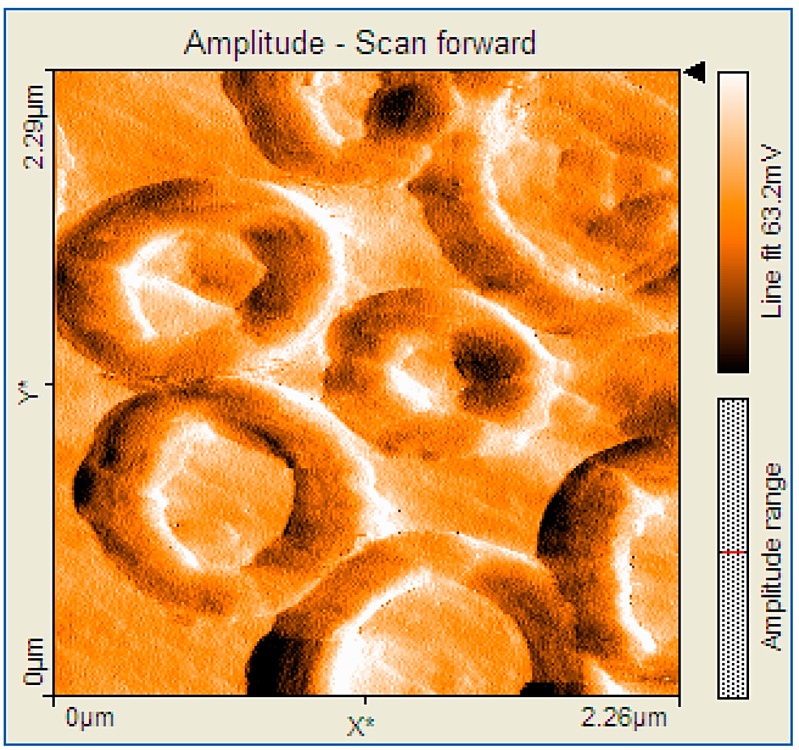
2D AFM image (2.2 × 2.2 µm) of porphyrin (PyTDMeOPP) ring architectures—single deposition from CHCl_3_.

Regarding 5-(4-pyridyl)-10,15,20-tris(3,4-dimethoxyphenyl)porphyrin, the initially formed triangular sandwich structures serve as building blocks for further aggregation into ring architectures (Figure 5 and Figure 6). The samples were formed by THF (or CHCl_3_) evaporation from a solution containing the porphyrin on silica plates. The orientation and aggregation of porphyrin (PyTDMeOPP) at the air–THF and air–CHCl_3_ interfaces was studied. 

Aggregation of porphyrin (PyTDMeOPP) at the air–THF interface gave triangular type morphology (2D AFM images, Figure 5a,b) with size in the range from 400 to 500 nm. These homogeneous triangles are randomly distributed, but uniformly oriented and very stable. If the deposition was made by multiple drop-casting operations, followed by evaporation of the solvent, a network of triangles of uniform size have been created by means of J type aggregation and a porous structure was obtained. Finally, this porous network is reorganized in more densely distributed ring type structures, similar with that presented in Section 2.8 (Figure 8), remaining stable even in its dry state on silica pure plates.

The nature of the solvent influences the architecture (Figure 6); when the deposition was made from CHCl_3,_ ring structures ranging in their internal diameter from 300 nm to 1 µm, but with the same width of the corona circular of approx. 200 nm were obtained.

### 2.7. Preliminary Tests of Porphyrin (PyTDMeOPP) for CO_2_ Detection

Recently, optical CO_2_ sensors based on the absorbance of pH indicator dye have been developed [4]. In our particular case, an optical CO_2_ sensor with the combination of colorimetric change of pH indicator dye will be developed. In order to decide if 5-(4-pyridyl)-10,15,20-tris(3,4-dimethoxyphenyl)porphyrin is appropriate for the formulation of CO_2_ sensors, some preliminary tests have been done to see its absorption intensity response to CO_2_ exposure and to establish the basis of an UV-vis sensor based on porphyrin for CO_2_ in water. A 1/1 THF/water solution of 3 × 10^−5^ mol/L of porphyrin (PyTDMeOPP), in a 50 mL vessel was prepared. A CO_2_ gas stream was produced by controlling the flow rates of CO_2_ and nitrogen gases. All the experiments are carried out at room temperature.

**Figure 7:**
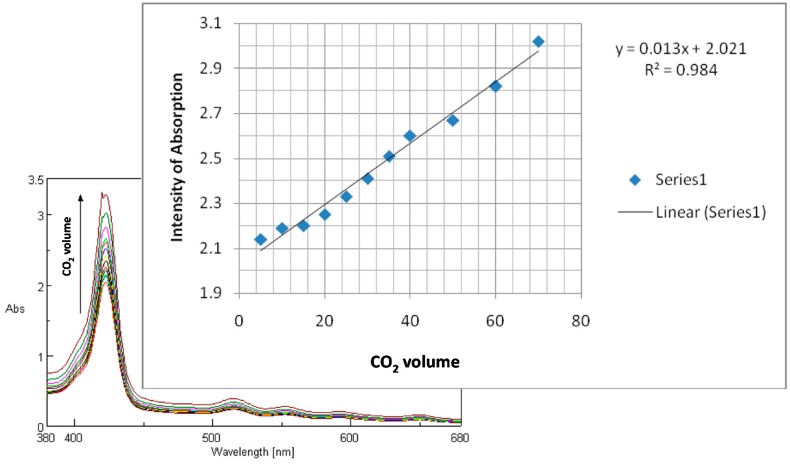
Dependence between increasing concentration of CO_2_ in water and the intensity of absorption of (PyTDMeOPP).

By introducing CO_2_ gas, the intensity of absorption of Soret band is increased and this effect is accompanied by the broadening of the Soret band. A linear dependence between absorption intensity measured at 423 nm and the increasing concentration of CO_2_ in water is observed (Figure 7).

A very good correlation coefficient of 98.4% was obtained for the linear dependence. Further work will focus on single wall carbon nanotube (SWCNT)—porphyrin composites as absorbing materials to improve the performance of the CO_2_ sensors [29].

### 2.8. Comparatively AFM Studies of the Porphyrin Morphology before and after CO_2_ Absorption

**Figure 8:**
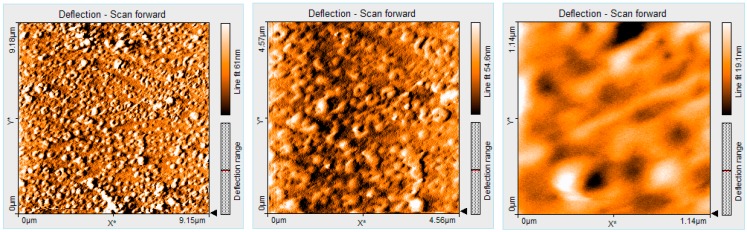
2D AFM images of ring aggregates of porphyrin in THF/water, before CO_2_ introduction.

The study of the porphyrin aggregates deposited on silica from the initial solution of THF/water shows the formation of the ring architectures (Figure 8), this phenomenon being discussed in detail in Section 2.6.

**Figure 9:**
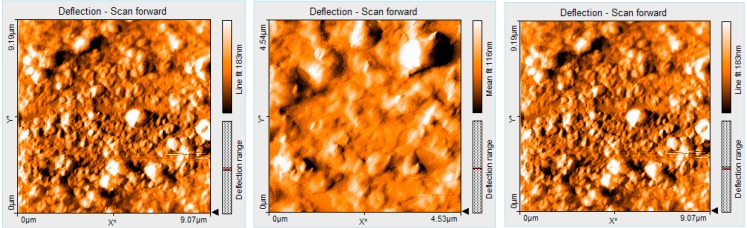
2D AFM images of ring aggregates of porphyrin (PyTDMeOPP) in THF/water, after 15 min of CO_2_ introduction.

After CO_2_ was introduced into the porphyrin (PyTDMeOPP) solution, the aggregate architecture changes significantly into amorphous deposits and all the inner parts of the porphyrin rings looked as if filled (Figure 9).

In order to highlight this statement, AFM measurements reveal that the surface roughness (Sa) is decreased from 81 nm, measured before CO_2_ interference (Figure 8 for 1.1 × 1.1 µm), to 5.5 nm, measured after 15 min of CO_2_ introduction (Figure 9 for the same size 1.1 × 1.1 µm).

## 3. Experimental Section 

### 3.1. Apparatus and Reagents 

FT-IR spectra were recorded on a JASCO 430 FT-IR apparatus (Easton, PA, USA), in the 4000–400 cm^−1^ range. Samples were prepared as KBr pellets. UV-visible spectra were recorded on a LAMBDA 12 UV/VIS spectrometer (Perkin Elmer, Boston, MA, USA) and on a JASCO model V-650 UV-visible spectrometer (Tokyo, Japan). Fluorescence spectra were obtained on a Perkin Elmer Model LS 55 apparatus (Boston, MA, USA). ^1^H-NMR spectra were registered on a 400 MHz Bruker spectrometer (Delft, Netherland), in CDCl_3_. The chemical shifts are expressed in δ (ppm). Proton chemical shifts were internally referenced to the residual proton resonance in CDCl_3_ (δ 7.26). The HPLC analyses were performed on a JASCO apparatus (Cremella, Italy) equipped with a KROMASIL 100 SI 5 µm polar column, 240 × 4 mm with a MD 1510 detector, at ambient temperature, using UV detection at 420 nm. The samples were subjected to analysis (20 μL) at a flow rate of 1 mL/min with acetone/toluene of ratio 1/1 as eluent. A Bruker Esquire HCT series mass spectrometer (Daltonics, Bremen, Germany) with Atmospheric Pressure Interface-Electro Spray Ionization was used for recording MS. 

#### 3.1.1. Spectroscopic Studies 

Absorption, excitation and emission spectra were registered at room temperature using 1 cm path length cells. The fluorescence spectra were recorded using slit widths, 4 for excitation and 3 for emission with 515 nm cut-off filters. Acidic conditions in THF solvent were changed by using 0.1 M HCl stock aqueous solutions. The pH values were measured with a digital Radelkis pH-meter (Budapest, Hungary). 

#### 3.1.2. Surface Imaging

Atomic force microscopy (AFM) investigations were carried out with Nanosurf^®^ EasyScan 2 Advanced Research AFM (Neuchatel, Switzerland). Samples were prepared onto a silica plate [30] from THF or CHCl_3_ solutions. All AFM measurements were done in contact mode or tapping mode, in environmental conditions (temperature: 20 ± 2 °C; relative humidity: 50%–70%).

All reagents were *p.a*. grade and were provided by Fluka (Basel, Switzerland), Aldrich (Taufkirchen, Germany) and Merck (Darmstadt, Germany) and used as received, excepting pyrrole, which was distilled prior to use. The solvents, such as chloroform were stored over 4 Å molecular sieves and CH_2_Cl_2_ was distilled from CaH_2_ under nitrogen. 

### 3.2. Method for Synthesis of 5-(4-Pyridyl)-10,15,20-tris-(3,4-dimethoxyphenyl)-porphyrin

Porphyrin (PyTDMeOPP) was synthesized by the multicomponent method [31,32,33,34,35,36] from a mixture of 3,4-dimethoxybenzaldehyde (3.375 g, 22.5 mmol) and isonicotinaldehyde (0.803 g, 7.5 mmol) dissolved in propionic acid (220 mL) and propionic anhydride (3.85 mL, 30 mmol), stirred at 100 °C. Then, pyrrole (2.13 mL, 30 mmol) mixed with propionic acid (5 mL) was slowly added. The resulting mixture was allowed to reflux for 90 min at 141 °C, further it was cooled to room temperature and then it was poured into ethyl ether (200 mL). No oxidant was added to the reaction mixture and the six mixed porphyrins were directly obtained in their oxidized form by using atmospheric O_2_. The precipitated material (dark-violet mass) was collected by vacuum filtration.

### 3.3. Separation Procedure

The solid product, which is a mixture of six porphyrins (two symmetrical, two unsymmetrical A_3_B and AB_3_ and the *cis* and *trans* isomers of A_2_B_2_ structure) was washed repeatedly with hot water, in 100 mL portions, until the rinse solution was no longer dark. The precipitate was dried under vacuum at 120 °C for 6 hours to remove propionic acid. 

The purple product, isolated from polypyrrolic side-reaction compounds, was subjected to HPLC on preparative polar Kromasil column using as eluent acetone/toluene 1/1 and porphyrin (PyTDMeOPP) gave its signal at 4.680 min (assigned after NMR and MS). 

### 3.4. The Main Characteristics of 5-(4-Pyridyl)-10,15,20-tris(3,4-dimethoxyphenyl)porphyrin

Dark violet crystalline solid; yield: 10.1% mp > 320 °C: FT-IR (KBr, ν, cm^−1^): 754 and 796 (γ C-H_Pirol_), 1017, 1232 and 1250 (aromatic C-O-CH_3_), 1507 (C-O-CH_3_ and/or ν C=N), 1587(ν C=C_Ph_), 2830 and 2927 (ν C-H), 3311 (ν N-H); ^1^H-NMR (400 MHz, CDCl_3_): 8.91 (6H, s, β pyrrole ), 8.81 (2H, s, β pyrrole), 8.20 (2H, s, 3,5-pyridyl), 7.78–7.80 (3H, m, 5-phenyl), 7.27–7.25 [8H, m, (6H, 2,6-phenyl and 2H, 2,6-pyridyl)], 4.18 (9H, bs, 4-methoxy), 3.99 (9H, bs, 3-methoxy), −2.78 (2H, bs, internal pyrrole); UV-vis, THF (λ_max_ (log ε)): 423.2 (5.58); 516.5 (4.38); 553.2 (4.14); 593.3 (3.90); 650.3 (3.77); HPLC (KROMASIL column, acetone/toluene = 1/1), R_T_, min: 4.680; MS (ESI^+^): *m/z* = 796.2 [M]^+^ (C_49_H_41_N_5_O_6_)^+^ molecular ion, calcd for [M]^+^795.87. 

## 4. Conclusions

A new nanostructured material based on a mixed substituted asymmetrical A_3_B porphyrin, namely: 5-(4-pyridyl)-10,15,20-tris(3,4-dimethoxyphenyl)-porphyrin (PyTDMeOPP) was synthesized by multicomponent reaction and characterized by ^1^H-NMR, FT-IR, UV-vis, fluorescence, MS, HPLC and AFM techniques. Introducing one pyridyl substituent in the 5-*meso*-position of porphyrin macrocycle confers some degree of hydrophilicity and may cause amazing self-assembly properties and a better response to increased acidity. The influence of pH and nature of the used solvent upon H and J aggregates of the porphyrin were discussed. Aggregation of porphyrin (PyTDMeOPP) at the air–THF interface gave a triangular type morphology, randomly distributed but uniformly oriented. If the deposition was made by multiple drop-casting operations, followed by evaporation of the solvent, a network of triangles of uniform size has been created and a porous structure was obtained, finally being reorganized into rings. When the deposition was made from CHCl_3,_ ring structures ranging in internal diameter from 300 nm to 1 µm, but with the same width of the corona circle of approx. 200 nm were obtained. This porphyrin-based material, capable of generating ring type architectures in both THF and CHCl_3_, has been proven to be sensitive to CO_2_. The linear dependence between the intensity of the porphyrin UV-vis absorption at a wavelength of 423 nm and the concentration of CO_2_ has a good correlation of 98.4%. In order to develop a sensor for CO_2_ detection, further work will be done to study the use of SWCNs and (PyTDMeOPP), and all the steps for validation of the sensor will be performed. 

## References

[1] Amao Y., Nakamura N. (2004). Optical CO_2_ sensor with the combination of colorimetric change of α-naphtholphthalein and internal reference fluorescent porphyrin dye. Sens. Actuators B.

[2] Amao Y., Komori T., Nishide H. (2005). Rapid responsible optical CO_2_ sensor of the combination of colorimetric change of α-naphtholphthalein in poly(trimethylsiliylpropyne) layer and internal reference fluorescent porphyrin in polystyrene layer. React. Funct. Polym..

[3] Amao Y., Komori T. (2005). Optical CO_2_ sensor of the combination of colorimetric change of α-naphtholphthalein in poly(isobutyl methacrylate) and fluorescent porphyrin in polystyrene. Talanta.

[4] Amao Y., Nakamura N. (2005). An optical sensor with the combination of colorimetric change of α-naphtholphthalein and internal reference luminescent dye for CO_2_ in water. Sens. Actuators B.

[5] Borchert N.B., Kerry J.P., Papkovsky D.B. (2013). A CO_2_ sensor based on Pt-porphyrin dye and FRET scheme for food packaging applications. Sens. Actuators B.

[6] Perez de Vargas-Sansalvador I.M., Carvajal M.A., Roldán-Muñoz O., Banqueri J., Fernández-Ramos M., Capitán-Vallvey L. (2009). Phosphorescent sensing of carbon dioxide based on secondary inner-filter quenching. Anal. Chim. Acta.

[7] Carvajal M.A., Pérez de Vargas-Sansalvador I.M., Palma A.J., Fernández-Ramos M.D., Capitán-Vallvey L.F. (2010). Hand-held optical instrument for CO2 in gas phase based on sensing film coating optoelectronic elements. Sens. Actuators B.

[8] Bevilaqua R.C.A., Zanella I., Fagan S.B. (2010). Chlorophyll a and pheophytin a as gas sensors of CO_2_ and O_2_ molecules. Chem. Phys. Lett..

[9] Gu C., Sun L., Zhang T., Li T. (1996). The design and characteristics of a porphyrin LB film ChemFET gas sensor. Thin Solid Films.

[10] Arnold D.P., Manno D., Micocci G., Serra A., Tepore A., Valli L. (1998). Gas-sensing properties of porphyrin dimer Langmuir-Blodgett films. Thin Solid Films.

[11] Popescu M., Simandan I.D., Sava F., Velea A., Fagadar-Cosma E. (2011). Sensor of nitrogen dioxide based on single wall carbon nanotubes and manganese-porphyrin. Dig. J. Nanomater. Biostructures.

[12] Cristescu R., Popescu C., Popescu A.C., Grigorescu S., Mihailescu I.N., Ciucu A.A., Iordache S., Andronie A., Stamatin I., Fagadar-Cosma E. (2011). MAPLE deposition of Mn(III) metalloporphyrin thin films: Structural, topographical and electrochemical investigations. Appl. Surf. Sci..

[13] Sivalingam Y., Magna G., Pomarico G., Catini A., Martinelli E., Paolesse R., di Natale C. (2013). The light enhanced gas selectivity of one-pot grown porphyrins coated ZnO nanorods. Sens. Actuators B.

[14] Li Y.W., Yao J.H., Liu C.J., Yang J.W., Yang C.L. (2009). Theoretical investigation of the O_2_ adsorption effect in the electron transport of single Fe-porphyrin molecule. Phys. Lett. A.

[15] Baschir L., Fagadar-Cosma E., Creanga I., Palade A., Lascu A., Birdeanu M., Savastru D., Savu V., Antohe S., Velea A. (2014). UV sensing effect in Langmuir-Blodgett complex films containing a novel synthesized Fe(III) porphyrin. Dig. J. Nanomater. Biostructures.

[16] Tripathi V.S., Lakshminarayana G., Nogami M. (2010). Optical oxygen sensors based on platinum porphyrin dyes encapsulated in ORMOSILS. Sens. Actuators B.

[17] Frankenberg C., O’Dell C., Berry J., Guanter L., Joiner J., Köhler P., Pollock R., Taylor T.E. (2014). Prospects for chlorophyll fluorescence remote sensing from the Orbiting Carbon Observatory-2. Remote Sens. Environ..

[18] Fagadar-Cosma E., Enache C., Vlascici D., Fagadar-Cosma G., Vasile M., Bazylak G. (2009). Novel nanomaterials based on 5,10,15,20-tetrakis(3,4-dimethoxyphenyl)-21*H*,23*H*-porphyrin entrapped in silica matrices. Mater. Res. Bull..

[19] Zakavi S., Gharab N.G. (2007). Interaction of para-substituted *meso*-tetraphenylporphyrins and *meso*-tetra(*n*-propyl)porphyrin with weak and strong carboxylic acids: A UV-vis spectroscopic study. Polyhedron.

[20] McRae E.G., Kasha M. (1958). The enhancement of phosphorescence ability upon aggregation of dye molecules. J. Chem. Phys..

[21] Di Natale C., Monti D., Paolesse R. (2010). Chemical sensitivity of porphyrin assemblies. Mater. Today.

[22] Mihailescu G., Olenic L., Garabagiu S., Blanita G., Fagadar-Cosma E., Biris A. (2010). A coupling between plasmonic resonances in nanoparticles and porphyrins molecules. J. Nanosci. Nanotechnol..

[23] Mohnani S., Bonifazi D. (2010). Supramolecular architectures of porphyrins on surfaces: The structural evolution from 1D to 2D to 3D to devices. Coord. Chem. Rev..

[24] Grama S., Hurduc N., Fagadar-Cosma E., Vasile M., Tarabukina E., Fagadar-Cosma G. (2010). Novel porphyrin-based polysiloxane micromaterial. Dig. J. Nanomater. Biostructures.

[25] Fagadar-Cosma E., Enache C., Fagadar-Cosma G., Savii C. (2007). Design of hybrid nanomaterials based on silica-porphyrin. AFM characterization. J. Optoelectron. Adv. Mater..

[26] Cristescu R., Popescu C., Popescu A.C., Mihailescu I.N., Ciucu A.A., Andronie A., Iordache S., Stamatin I., Fagadar-Cosma E., Chrisey D.B. (2010). Functional porphyrin thin films deposited by matrix assisted pulsed laser evaporation. Mater. Sci. Eng. B-Adv..

[27] Teixeira R., Andrade S.M., Vaz Serra V., Paulo P.M.R, Sánchez-Coronilla A., Neves M.G.P.M.S., Cavaleiro J.A.S., Costa S.M.B. (2012). Reorganization of Self-Assembled Dipeptide Porphyrin J-Aggregates in Water–Ethanol Mixtures. J. Phys. Chem. B.

[28] Vaz Serra V., Andrade S.M., Neves M.G.P.M.S., Cavaleiro J.A.S., Costa S.M.B. (2010). J-aggregate formation in bis-(4-carboxyphenyl)porphyrins in water: pH and counterion dependence. New J. Chem..

[29] Lvova L., Mastroianni M., Pomarico G., Santonico M., Pennazza G., di Natale C., Paolesse R., D’Amico A. (2012). Carbon nanotubes modified with porphyrin units for gaseous phase chemical sensing. Sens. Actuators B.

[30] Fagadar-Cosma E., Enache C., Dascalu D., Fagadar-Cosma G., Gavrila R. (2008). FT-IR, fluorescence and electronic spectra for monitoring the aggregation process of tetra-pyridylporphyrine entrapped in silica matrices. Optoelectron. Adv. Mater..

[31] Adler A.D., Longo F.R., Goldmacher J., Assour J., Korsakoff L. (1967). A simplified synthesis for *meso*-tetraphenylporphine. J. Org. Chem..

[32] Lindsey J.S., Schreiman I.C., Hsu H.C., Kearney P.C., Marguerettaz A.M. (1987). Rothemund and Adler-Longo reactions revisited: Synthesis of tetraphenylporphyrins under equilibrium conditions. J. Org. Chem..

[33] Fazekas M., Pintea M., Senge M.O., Zawadzka M. (2008). Synthetic strategies and porphyrin building blocks for unsymmetrical multichromophores. Tetrahedron Lett..

[34] Marek D., Narra M., Schneider A., Swavey S. (2006). Synthesis, characterization and electrode adsorption studies of porphyrins coordinated to ruthenium(II) polypyridyl complexes. Inorg. Chim. Acta.

[35] Lindsey J.S. (1991). Self-assembly in synthetic routes to molecular devices. Biological principles and chemical perspectives: A review. New. J. Chem..

[36] Fagadar-Cosma E., Cseh L., Badea V., Fagadar-Cosma G., Vlascici D. (2007). Combinatorial synthesis and characterization of new asymmetric porphyrins as potential photosensitizers in photodynamic therapy. Comb. Chem. High Throughput Screen..

